# The polyomaviruses WUPyV and KIPyV: a retrospective quantitative analysis in patients undergoing hematopoietic stem cell transplantation

**DOI:** 10.1186/1743-422X-9-209

**Published:** 2012-09-18

**Authors:** Nasim Motamedi, Helga Mairhofer, Hans Nitschko, Gundula Jäger, Ulrich H Koszinowski

**Affiliations:** 1 Max von Pettenkofer-Institute, Ludwig-Maximilians-University, Department of Virology, Pettenkoferstr. 9a, Munich D-80336, Germany

**Keywords:** WU polyomavirus, KI polyomavirus, Gastrointestinal Tract, Respiratory system, Urine, Hematopoietic Stem Cell Transplantation, Immunocompromised Host, Viral Load

## Abstract

**Background:**

The polyomaviruses WUPyV and KIPyV have been detected in various sample types including feces indicating pathogenicity in the gastrointestinal (GI) system. However, quantitative viral load data from other simultaneously collected sample types are missing. As a consequence, primary replication in the GI system cannot be differentiated from swallowed virus from the respiratory tract.

Here we present a retrospective quantitative longitudinal analysis in simultaneously harvested specimens from different organ sites of patients undergoing hematopoietic stem cell transplantation (HSCT). This allows the definition of sample types where deoxyribonucleic acid (DNA) detection can be expected and, as a consequence, the identification of their primary replication site.

**Findings:**

Viral DNA loads from 37 patients undergoing HSCT were quantified in respiratory tract secretions (RTS), stool and urine samples as well as in leukocytes (n = 449). Leukocyte-associated virus could not be found. WUPyV was found in feces, RTS and urine samples of an infant, while KIPyV was repeatedly detected in RTS and stool samples of 4 adult patients.

RTS and stool samples were matched to determine the viral load difference showing a mean difference of 2.3 log copies/ml (p < 0.001).

**Conclusions:**

The data collected in this study suggest that virus detection in the GI tract results from swallowed virus from the respiratory tract (RT). We conclude that shedding from the RT should be ruled out before viral DNA detection in the feces can be correlated to GI symptoms.

## Findings

### Background

In 1971, the first human pathogenic polyomaviruses (PyV) BKV and JCV were discovered in severely immunosuppressed patients [1,2]. Since then, BKV and JCV have been detected in various sample types from different organ systems including urine specimens, serum samples and leukocytes, which seem to form the vehicle for virus spread [3-6].

The two polyomaviruses WU polyomavirus (WUPyV) and KI polyomavirus (KIPyV) were first described in 2007 [7,8]. Recent findings imply that primary contact occurs during childhood resulting in a persistent infection [9-11]. They have mainly been detected in respiratory tract (RT) secretions (RTS) but also in stool samples [7,12-17], raising the question of a causal connection to gastrointestinal (GI) symptoms [13-16,18]. So far, however, no clear association between virus detection and specific symptoms could be shown. Like previously described for BKV/JCV, an association between the appearance of WUPyV/KIPyV and immunosuppression – especially with hematopoietic stem cell transplantation (HSCT) - has repeatedly been observed [9,13,14,19]. In analogy to findings of BKV and JCV in leukocytes [3-6], a hematologic reservoir is also suspected for WUPyV and KIPyV [16].

To elucidate the tissue tropism of these viruses many studies followed a cross-sectional design. Such studies, however, have not revealed significantly different prevalence rates for patients and control groups [20,21]. Hence, as seen for BKV/JCV, quantitative assignment of viral loads could be indicative for clinically relevant viral replication [22]. This theory is supported by a recent study reporting higher KIPyV loads in RTS of patients undergoing HSCT than of the control group [20].

As mentioned, WUPyV/KIPyV DNA has been detected in stool samples. However, a common challenge when detecting viral DNA in the GI tract is the differentiation between possibly pathogenic primary replication and secondary shedding due to swallowed virus [23]. This problem can only be addressed by longitudinal viral DNA quantification in different simultaneously collected sample types.

Therefore, we performed this retrospective analysis to provide quantitative viral load data in different specimens of patients undergoing HSCT. Furthermore, we address the question of genuine viral replication in the gastrointestinal tract as opposed to detection of viral DNA by polymerase chain reaction (PCR) due to secondary shedding from the respiratory system.

### Material and methods

A retrospective analysis for the prevalence of WUPyV and KIPyV in HSCT-patients was performed with a total of 449 clinical samples from 37 HSCT-patients. These patients were routinely screened for viral pathogens throughout HSCT-therapy, which comprised WUPyV and KIPyV as well as EBV, CMV, HHV6 and Adenovirus. In case of a clinical suspicion, further PCR testing was performed for HSV, Norovirus, Rotavirus, Parvovirus B19, Influenza virus and Parainfluenza virus.

The study was conducted in accordance with the Declaration of Helsinki. All patients gave informed consent to virological surveillance, collection and publication of data. Approval was waived by the ethics committee of the Ludwig-Maximilians-University.

Assays were carried out according to standard detection techniques of the accredited laboratory for virological diagnostics of the Max von Pettenkofer-Institute.

The study comprised RTS (throat washes, bronchoaleveolar lavages, endotracheal suctions), stool and urine samples and leukocytes. Samples were stored at 4°C and nucleic acid was extracted within 24 h with the High Pure Viral Nucleic Acid Kit (Roche Applied Biosystems). Leukocytes were adjusted to a cell number of 20,000 cells/assay. Quantitative real time-PCR was performed as previously described [24] as an *in house* test with slightly modified primers: 5’-TTGGATGAAAATGGCATTGG-3’, 5’-AACCCTTCTTTGTCTAAARTGTAGCC-3’; Probes: WU: 5’-FAM-CATAACTTGTGCTGACCTTTTGGGAGTTAAC-BHQ1-3’, KI: 5’-HEX-ACATTACTTGTGCAGATATGCTTGGAACAGC-BHQ1-’3 (ELLA Biotech GmbH, Martinsried, Germany). Positive control plasmids for WUPyV and KIPyV were kindly provided by Benedikt Weißbrich (University of Würzburg, Germany).

The sensitivity threshold of the assay with a detection probability of 90% was 7 copies/20,000 cells for leukocytes and was 350 copies/ml for RTS, stool and urine samples, corresponding to 2.54 log copies/ml.

RTS and stool samples that tested positive were matched to define the mean difference between viral loads in RTS and stool samples.

Our statistical inference regarding the differences in log viral loads was based on a two-sided paired t test. A p value < 0.05 was regarded as significant.

### Results

#### Initial analysis

During the initial analysis, 19 out of 208 specimens taken from 37 patients tested positive for WUPyV or KIPyV. Positive results could be assigned to 5 patients (total number of samples from these 5 patients: 64) (Table1). KIPyV was detectable in RTS and stool samples from 4 adult patients, whereas WUPyV was found in RTS, stool and urine samples of an infant.

**Table 1:** Characteristics of the 5 hematopoietic stem cell transplantation (HSCT) patients presenting WUPyV or KIPyV

**Virus**	**Patient**	**Transplant type**	**GvHD**	**Antiviral therapy**	**Co-infecting viruses in RTS**	**Co-infecting viruses in stool**	**Co-infecting viruses in further samples**
**KIPyV (adult patients)**	1	Double Cord-Blood- Transplantation	Chronic (skin, gut - max. IV°)	Cidofovir (days +121 to +272)	HHV6, HSV	HHV6	HHV6 (leukocytes), BKV, HHV6 (urine)
	2	Allogeneic PBSCT	Chronic ( skin, gut - max. IV°)	Cidofovir, Ribavirin ( days +118 to +176)	EBV, HHV6, HSV	Adenovirus, EBV	EBV, HHV6, Adenovirus (leukocytes)
	3	Allogeneic BMT	Acute (skin - max. II°)	Foscarnet ( days +19 to +55), Cidofovir ( days +55 to +138)	HHV6, EBV	none	EBV (leukocytes)
	4	Allogeneic PBSCT	Acute (gut - max. III°)	none	HHV6	HHV6	HHV6, EBV (leukocytes)
**WUPyV (infant)**	5	Allogeneic PBSCT	Chronic (pre-PBSCT: skin, gut, hematopoietic system - max. III°)	Cidofovir ( days +17 to +58)	HHV6, Adenovirus, HHV7, HSV	Norovirus, Adenovirus, HHV6	HHV6 (urine)

Clinical data summary for the HSCT patients that tested positive for WUPyV and KIPyV including detected co-infecting viruses throughout the observation period and antiviral therapy; Antiviral therapy: all patients received prophylactic Acyclovir therapy. Further antiviral therapy is depicted with time-points (day 0: day of HSCT). Co-infecting viruses in RTS, stool and further samples in order of detection frequency. GvHD: Graft versus Host Disease; RTS: respiratory tract samples, PBSCT: peripheral blood stem cell transplantation.

#### Follow-Up

**Figure 1:**
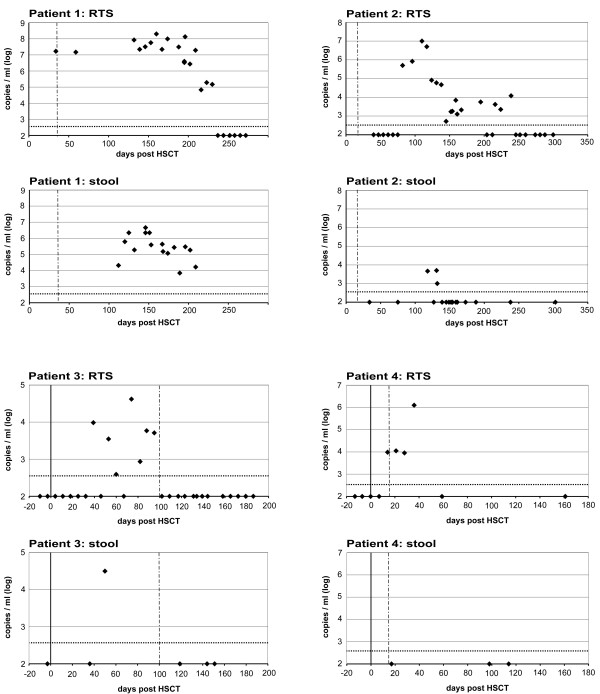
**Courses of KIPyV load in respiratory tract samples and stool samples in 4 adult patients.** Quantitative Real Time-PCR was performed to determine viral loads (copies/ml) in different specimens before and after hematopoietic stem cell transplantation (HSCT). The 90% sensitivity threshold was at 350 copies/ml corresponding to 2.54 log copies/ml indicated by the horizontal dotted line. The bold line represents day 0 of HSCT; the dashed vertical line shows time point of neutrophil granulocyte engraftment. Please note that most samples were collected after HSCT. RTS: respiratory tract secretion.

**Figure 2:**
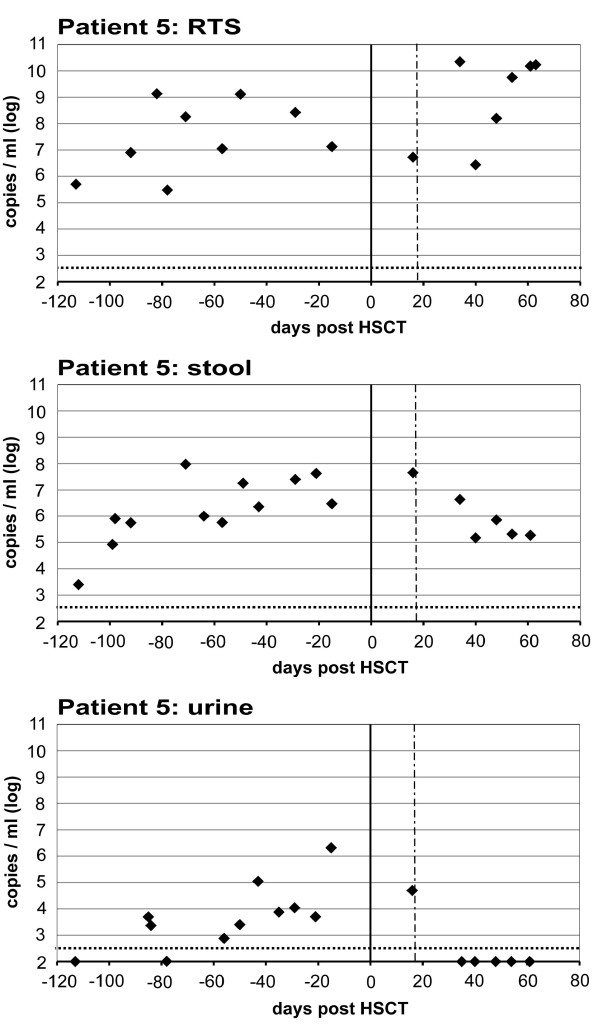
**Courses of WUPyV viral load in respiratory tract samples, stool and urine samples in infant patient 5.** Quantitative Real Time-PCR was performed to determine viral loads (copies/ml) in different specimens before and after hematopoietic stem cell transplantation (HSCT). The 90% sensitivity threshold was at 350 copies/ml corresponding to 2.54 log copies/ml indicated by the horizontal dotted line. The bold line represents day 0 of HSCT; the dashed vertical line shows time point of neutrophil granulocyte engraftment. RTS: respiratory tract secretion.

In these 5 patients, viral loads were defined over time in another 241 samples from different organ sites (RTS, stool, leukocytes, urine; total: 305 samples) (Figure 1,2). All patients had a period of diarrhea during HSCT which coincided with detection of WUPyV/KIPyV, but also with mucositis and Graft versus Host Disease (GvHD – stages were defined according to Glucksberg et al. [25]) of the gut and detection of co-infecting pathogenic viruses (Table1). Thus, primary and possibly pathogenic replication in the gastrointestinal tract needed to be differentiated from secondary shedding events from the RT. For this purpose, RTS and stool specimens collected with a maximum time difference of 24 hours were matched in pairs (n = 34) and viral load differences were defined. A significant mean viral load difference of 2.3 log copies/ml was determined between these 2 sample types (p < 0.001).

Repeated testing of leukocytes showed neither KIPyV nor WUPyV. Leukocyte-associated viremia was therefore not seen in these patients despite simultaneous detection of high viral titers in other specimens and the severe immunosuppression throughout HSCT-therapy.

A pediatric patient presented WUPyV in feces and RTS as well as in urine samples (Figure2). Previous studies indicate that primary infection with WUPyV/KIPyV occurs during childhood [9-11]. Thus, this could have been a case of primary infection for WUPyV which led to virus detection in the urine with lower viral loads than in RTS and stool samples.

KIPyV was not detectable in urine samples at any time point.

Prophylactic therapy with Acyclovir was routinely performed for all patients under HSCT. Further antiviral therapy is depicted in Table1. All patients presented HHV6, among other co-infecting viruses (Table1).

Patients 1 and 2 had chronic Graft versus Host Disease (maximum WHO stage 4) until the end of the observation period that was treated with high doses of various immunosuppressants including Infliximab. Patient 1 developed a BKV-associated hemorrhagic cystitis. For this reason and for the HHV6-infection (Table1), antiviral therapy with Cidofovir was initiated on day +121 and lasted till the end of observation. While he could not clear BKV throughout the course of analysis, he did clear KIPyV (Figure1).

Patient 2 was treated with Cidofovir and Ribavirin from day +118 to +176. From day +71 to +99 he had received 5 doses of Infliximab. The first time-point of virus detection was at day +82, followed by a steady decline and clearing of KIPyV until day + 246 (Figure1).

Patient 3 was treated with Foscarnet and Cidofovir for HHV6 and EBV (Table1) from day +55 to +138. Clearing of virus coincided with antiviral treatment and neutrophil engraftment.

Patient 4 did not receive further antiviral therapy and cleared the virus after a viral load peak of 6.1 log copies/ml 21 days after neutrophil engraftment.

Patient 5 presented WUPyV among various co-infecting viruses. For an adenoviral infection, he was treated with Cidofovir from day +17 to +58, which coincides with clearing of the virus from the urine, but not in RTS or stool samples.

### Conclusions

In this retrospective longitudinal study, a total of 449 samples (RTS, stool, urine, leukocytes) from 37 HSCT-patients were quantitatively analyzed for the polyomaviruses WUPyV and KIPyV. KIPyV was found in RTS and stool samples of four adult patients, while an infant presented WUPyV in RTS and stool samples as well as in urine.

Recent studies raised the question of a causal connection between WUPyV/KIPyV detection in the stool and the appearance of GI symptoms [13-16,18]. In this study, viral loads in RTS and stool samples correlated consistently. A mean viral load difference of 2.3 log copies/ml (p < 0.001) between respiratory and stool samples was observed. This suggests the RT as the primary site of replication with virus detection in feces resulting from swallowed virus. We conclude that, when screening for WUPyV or KIPyV in stool samples to define associated symptoms, respiratory secretions should be simultaneously analyzed to rule out viral shedding from the respiratory tract.

These polyomaviruses were repeatedly detected in all 5 patients and viral load courses could be quantified. Clearing of KIPyV coincided with Cidofovir therapy in patients with severe immunosuppressive therapy due to high-grade GvHD and with immune reconstitution as determined by neutrophil granulocyte engraftment in patients with moderate immunosuppressive therapy, respectively. Cidofovir is a treatment option for BKV [4], and might therefore have antiviral activity on related viruses, such as WUPyV or KIPyV. Therefore, more studies are needed to exclude or to specify a possible impact of this substance and to further define factors associated with viral replication taking into account clinical symptoms as well as medication and immune status.

## Competing interests

The authors declare that they have no competing interests.

## Authors’ contributions

NM was involved in study design, PCR analysis, interpretation of PCR and clinical data and wrote the manuscript. NM, HM and HN designed PCRs and performed PCR analysis. GJ and UK were involved in study design and interpretation of data. All authors read and approved the final manuscript.

## References

[B1] Gardner SD Field AM Coleman DV Hulme B New human papovavirus (B.K.) isolated from urine after renal transplantation Lancet 1971 1 1253 1257 4104714 10.1016/s0140-6736(71)91776-4

[B2] Padgett BL Walker DL ZuRhein GM Eckroade RJ Dessel BH Cultivation of papova-like virus from human brain with progressive multifocal leucoencephalopathy Lancet 1971 1 1257 1260 4104715 10.1016/s0140-6736(71)91777-6

[B3] Weber T Major EO Progressive multifocal leukoencephalopathy: molecular biology, pathogenesis and clinical impact Intervirology 1997 40 98 111 10.1159/000150537 9450227

[B4] Ahsan N Shah KV Polyomaviruses and human diseases Adv Exp Med Biol 2006 577 1 18 10.1007/0-387-32957-9_1 16626024

[B5] Major EO Amemiya K Tornatore CS Houff SA Berger JR Pathogenesis and molecular biology of progressive multifocal leukoencephalopathy, the JC virus-induced demyelinating disease of the human brain Clin Microbiol Rev 1992 5 49 73 1310438 10.1128/cmr.5.1.49 PMC358223

[B6] Dorries K Vogel E Gunther S Czub S Infection of human polyomaviruses JC and BK in peripheral blood leukocytes from immunocompetent individuals Virology 1994 198 59 70 10.1006/viro.1994.1008 8259683

[B7] Allander T Andreasson K Gupta S Bjerkner A Bogdanovic G Persson MA Dalianis T Ramqvist T Andersson B Identification of a third human polyomavirus J Virol 2007 81 4130 4136 10.1128/JVI.00028-07 17287263 PMC1866148

[B8] Gaynor AM Nissen MD Whiley DM Mackay IM Lambert SB Wu G Brennan DC Storch GA Sloots TP Wang D Identification of a novel polyomavirus from patients with acute respiratory tract infections PLoS Pathog 2007 3 e64 10.1371/journal.ppat.0030064 17480120 PMC1864993

[B9] Abedi Kiasari B Vallely PJ Corless CE Al-Hammadi M Klapper PE Age-related pattern of KI and WU polyomavirus infection J Clin Virol 2008 43 123 125 10.1016/j.jcv.2008.05.003 18573691 PMC7108349

[B10] Nguyen NL Le BM Wang D Serologic evidence of frequent human infection with WU and KI polyomaviruses Emerg Infect Dis 2009 15 1199 1205 10.3201/eid1508.090270 19751580 PMC2815979

[B11] Kean JM Rao S Wang M Garcea RL Seroepidemiology of human polyomaviruses PLoS Pathog 2009 5 e1000363 10.1371/journal.ppat.1000363 19325891 PMC2655709

[B12] Bergallo M Terlizzi ME Astegiano S Ciotti M Babakir-Mina M Perno CF Cavallo R Costa C Real time PCR TaqMan assays for detection of polyomaviruses KIV and WUV in clinical samples J Virol Methods 2009 162 69 74 10.1016/j.jviromet.2009.07.016 19646480 PMC7119675

[B13] Babakir-Mina M Ciccozzi M Alteri C Polchi P Picardi A Greco F Lucarelli G Arcese W Perno CF Ciotti M Excretion of the novel polyomaviruses KI and WU in the stool of patients with hematological disorders J Med Virol 2009 81 1668 1673 10.1002/jmv.21559 19626610

[B14] Mourez T Bergeron A Ribaud P Scieux C de Latour RP Tazi A Socie G Simon F LeGoff J Polyomaviruses KI and WU in immunocompromised patients with respiratory disease Emerg Infect Dis 2009 15 107 109 10.3201/1501.080758 19116066 PMC2662633

[B15] Bialasiewicz S Whiley DM Lambert SB Nissen MD Sloots TP Detection of BK, JC, WU, or KI polyomaviruses in faecal, urine, blood, cerebrospinal fluid and respiratory samples J Clin Virol 2009 45 249 254 10.1016/j.jcv.2009.05.002 19515607

[B16] Babakir-Mina M Ciccozzi M Perno CF Ciotti M The novel KI, WU, MC polyomaviruses: possible human pathogens? New Microbiol 2011 34 1 8 21344140

[B17] Mueller A Simon A Gillen J Schildgen V Tillmann RL Reiter K Schildgen O Polyomaviruses KI and WU in children with respiratory tract infection Arch Virol 2009 154 1605 1608 10.1007/s00705-009-0498-2 19756357 PMC7087260

[B18] Neske F Blessing K Prottel A Ullrich F Kreth HW Weissbrich B Detection of WU polyomavirus DNA by real-time PCR in nasopharyngeal aspirates, serum, and stool samples J Clin Virol 2009 44 115 118 10.1016/j.jcv.2008.12.004 19157970 PMC7172993

[B19] Debiaggi M Canducci F Brerra R Sampaolo M Marinozzi MC Parea M Arghittu M Alessandrino EP Nava S Nucleo E Molecular epidemiology of KI and WU polyomaviruses in infants with acute respiratory disease and in adult hematopoietic stem cell transplant recipients J Med Virol 2010 82 153 156 10.1002/jmv.21659 19950241 PMC7166565

[B20] Rao S Garcea RL Robinson CC Simoes EA WU and KI polyomavirus infections in pediatric hematology/oncology patients with acute respiratory tract illness J Clin Virol 2011 52 28 32 10.1016/j.jcv.2011.05.024 21705268 PMC3816538

[B21] Norja P Ubillos I Templeton K Simmonds P No evidence for an association between infections with WU and KI polyomaviruses and respiratory disease J Clin Virol 2007 40 307 311 10.1016/j.jcv.2007.09.008 17997354 PMC7172997

[B22] Jiang M Abend JR Johnson SF Imperiale MJ The role of polyomaviruses in human disease Virology 2009 384 266 273 10.1016/j.virol.2008.09.027 18995875 PMC2661150

[B23] Schildgen O Muller A Simon A Human bocavirus and gastroenteritis Emerg Infect Dis 2007 13 1620 1621 18258026 10.3201/eid1310.070436 PMC2851507

[B24] Lindau C Tiveljung-Lindell A Goh S Ramqvist T Allander T A single-tube, real-time PCR assay for detection of the two newly characterized human KI and WU polyomaviruses J Clin Virol 2009 44 24 26 10.1016/j.jcv.2008.09.006 18980861

[B25] Glucksberg H Storb R Fefer A Buckner CD Neiman PE Clift RA Lerner KG Thomas ED Clinical manifestations of graft-versus-host disease in human recipients of marrow from HL-A-matched sibling donors Transplantation 1974 18 295 304 10.1097/00007890-197410000-00001 4153799

